# Expired Medication: Societal, Regulatory and Ethical Aspects of a Wasted Opportunity

**DOI:** 10.3390/ijerph17030787

**Published:** 2020-01-27

**Authors:** Faez Alnahas, Prince Yeboah, Louise Fliedel, Ahmad Yaman Abdin, Khair Alhareth

**Affiliations:** 1 Division of Bioorganic Chemistry, School of Pharmacy, Saarland University, 66123 Saarbruecken, Germany; faez.nahas1@gmail.com (F.A.); s8pryebo@stud.uni-saarland.de (P.Y.); s8ahabdi@stud.uni-saarland.de (A.Y.A.); 2 UTCBS (Chemical and Biological Technologies for Health Group), Faculté de Pharmacie de Paris, Université de Paris, CNRS, INSERM, 75006 Paris, France; louise.fliedel@parisdescartes.fr

**Keywords:** expiration date, expired medications, pharmaceutical waste, waste management

## Abstract

A massive volume of expired medications amasses annually around the world because of pharmaceutical overprescription, combined with overproduction. The accumulation of pharmaceutical waste imposes ecological, economic and social/ethical burdens. Managing this presumed “waste” has developed into a global challenge due to the absence of specific regulations, unreasonable behavior of the patients, and an improper understanding of the concept of “expired medications” in general. This paper summaries, first, the recent literature reporting practices related to the disposal of unused medications. In this context, 48 papers from 34 countries with a total of 33,832 participants point towards a significant lack of public awareness regarding the appropriate disposal of such biologically potent chemicals. These findings are corroborated by a local survey on the disposal practices of unused medicines among pharmacy students at Saarland University. The regulatory aspects surrounding this topic, often based on the official guidelines for the disposal of expired medications and local waste management strategies, are then discussed in light of these findings. Finally, a closer inspection of the epistemic values of expired medications and different strategies for managing expired medications have been reviewed.

## 1. Introduction

Medical advances during the last century have resulted in the dramatic increase in life expectancy and quality of life. This astonishing progress in healthcare has been accompanied by an increase in the volume of pharmaceutical waste, primarily due to the rise in the number of patients, prescriptions, consumption and overproduction of medications. Consequently, this pharmaceutical waste has resulted in ecological, economical and ethical burdens that need to be understood from different perspectives [1].

The Consumer Healthcare Products Association has reported that, over-the-counter medication (OTC) retail sales in the US have doubled between 2008 and 2018, from USD 16.8 bn to USD 35.2 bn [2]. Additionally, Variant Market Research, a data mining and information analysis company based in India, estimated that the universal OTC market is expected to grow from USD 125 bn in 2016 to USD 273 bn by 2024 [3]. In parallel, it has been reported by the German Federal Ministry for the Environment, that pharmaceutical companies in both the industrialized and the developing world are producing a variety of synthetic chemicals at a rate of 100,000 tons per year [4]. From this, only a fraction is being utilized whilst the greater portion eventually will be unused and/or expired resulting an important volume of pharmaceutical waste.

The impact of pharmaceutical waste is therefore multifaceted. In an economic sense, this waste entails a massive loss of financial resources. A cross-sectional study conducted in 2001 reported that the value of unused medications generated by the population of US senior citizens alone is estimated to be over USD 1 bn per year [5]. Similar situations have been observed in other developed countries. In Australia, the economic value of pharmaceutical waste per patient and year sums up to USD 1280 [6].

Besides the financial losses imposed on the healthcare systems of countries and on individual patients, the disposal of expired medications is equally problematic. These pharmaceutical products contain biologically active and often toxic substances, and therefore also threaten the ecosystem, especially if disposed inappropriately. The presence of these pharmaceuticals poses many risks on the aquatic environment. The U.S Geological Survey, for instance, discovered intersex fish in the Potomac River due to estrogen contamination [7]. In this context, another study realized by researchers in Canada detected 25 antibiotics in drinking water in small concentrations [8]. In addition, there are numerous studies indicating the presence of minute amounts of active pharmaceutical ingredients in the soil and waterways. These minute quantities of active drugs eventually enter the food chain, where they may actually become enriched again, and subsequently return—unintentionally—to the human population which had previously disposed of them [9]. 

The loss of expired medications is particularly frustrating as many developing countries suffer from an acute shortage of pharmaceuticals, such as antibiotics. Would it therefore be wise to redirect expired medications to the developing world, therefore avoiding “waste” on one side and shortages on the other?

In 1977, the World Health Organization (WHO) created a list for indispensable medications and recommended that countries should consider such medication as a type of national survival resource [10]. The WHO also created a depository for specific medications in case of emergencies. Whilst, a donation program aiming to redistribute unused medication in developing countries, could be a good initiative to avoid the waste of medication. The WHO, however, recommends in a guideline published in 1999 that expired or unused pharmaceuticals should never be reused [11].

The current handling of unused medication, including expired ones, has become a global canker. The disposal of pharmaceuticals by garbage and sewer is still the most common method in many countries with the absence of the proper disposal of expired medications from the patient side [12]. Besides, there is no specific outlined strategies for pharmaceutical waste from the legal and the healthcare authority side.

The objective of this study, then, is to provide a better understanding of the issue of “expired medications” from societal, regulatory and ethical perspectives. In this context, our areas of investigation can be summarized as follows: exploring the current situation about social behavior regarding proper medical disposal, listing the regulation to control medical disposal, and discussing the different strategies of waste management. In this sense, tracking the life cycle of expired medications will guide us to develop a discussion and to suggest some recommendations for appropriate disposal practices. 

In this paper, first, we have reviewed the recent literature on the current disposal methods. We, then, have presented the results of an empirical survey conducted at Saarland University, Germany, about individual disposal practices. Essential recommendations and regulations for proper medical disposal and different local waste management strategies have also been highlighted and commented on. Finally, we have developed a novel understanding of the concept of “expired medications”, which will emphasize their value and utilize their benefits.

## 2. Methods

### 2.1. Literature Review of Some Reported Disposal Practices around the World

We have reviewed previously reported disposal practices by the public around the world to obtain a better grasp on the general awareness regarding the issue of improper disposal methods.

Databases have been collected by using the keywords “medicine” or “medication” or drugs” and “unused” or “wastage” or “disposal” or “management” in different combinations in the search bar in Google Scholar, Knowledge TM, and PubMed up to the end of September 2019. Articles without an empirical investigation into disposal practices, through a public survey, were excluded from reviewed articles. Included papers were analyzed, results were collected and presented in 100% stacked bar using *Microsoft Excel^®^ 2016*. Countries were classified according to the Human Development Index (HDI) based on the report issued by the United Nations Development Programme (UNDP). More details about results are presented, also, in Table S1 in Supplementary Materials S1.

### 2.2. Local Survey at the Saarland University

To supplement these literature reports, a survey was conducted among students from Saarland University and their families to establish a baseline information on disposal practices.

A cross-sectional empirical study was conducted through face-to-face interviews using a pre-validated structured questionnaire (Supplementary Materials S2). A self-administered questionnaire was randomly provided to 140 undergraduate pharmacy students in March 2018 at Saarland University in Saarbruecken campus, Germany. In total, 88 valid questionnaires were collected, and the data was subsequently analyzed and presented in a pie chart using Microsoft Excel^®^ 2016. In addition, a statistical chi-squared test was applied to evaluate any difference between respondents based on both gender and education level.

The conducted questionnaire covered (i) the demographic characteristics of participants, i.e., age, gender and level of education; (ii) the consumption of prescription drugs; (iii) the disposal practices and a personal opinion of how to properly deal with unused medicines.

### 2.3. Regulations

The keywords “medicine” or “medication” or “drugs” and “unused” or “wastage” or “disposal” or “management” and “regulation” or “instructions” or “information” or “education” or “legislation” or “control” or “recommendations” and “WHO” or “Europe” or “FDA” or “Australia” or “Africa” or “Asia” were used in different combinations in the search bar in search engines, formal local resources, and official websites. All included databases were in English, German, or French, while those in other languages have been excluded.

### 2.4. Local Waste Management Strategies

To evaluate the impact on the environment from pharmaceuticals, we have explored the different regulations and strategies, around the world, for managing solid waste.

The keywords “garbage” or “waste” and “management” or “handling” or “control” and “Europe” or “America” or “Australia” or “Africa” or “Asia” or “global” were used in different combinations in the search bar in search engines, formal local resources, and other official websites.

## 3. Results

### 3.1. Literature Review of Some Reported Disposal Practices around the World

The improper disposal of unused medicines through environmentally unfavorable routes, such as in garbage or sewers, results in the presence of active pharmaceutical compounds in the ecosystem, leading to detrimental effects on human health and the environment as a whole [13,14].

There have been two methodical reviews on the topic of disposal methods of expired/unused medications. The first reviewed articles published from 1986 up to May 2010, and focused on the disposal methods of unused medicines around the world [12]; the second covered January 2005 up to September 2015 and considered the practice of medication disposal worldwide while aiming to obtain a deeper insight into a possible connection between environmental awareness and people’s behavior regarding this issue [15]. 

In this study, we reviewed 48 publications reporting surveys carried out in 34 countries, a total of 33,832 participants were involved. There were 13 studies conducted in Europe. Besides, Asia (11), Africa (7), North America (2), South America (2), and Australia (2).

**Figure 1:**
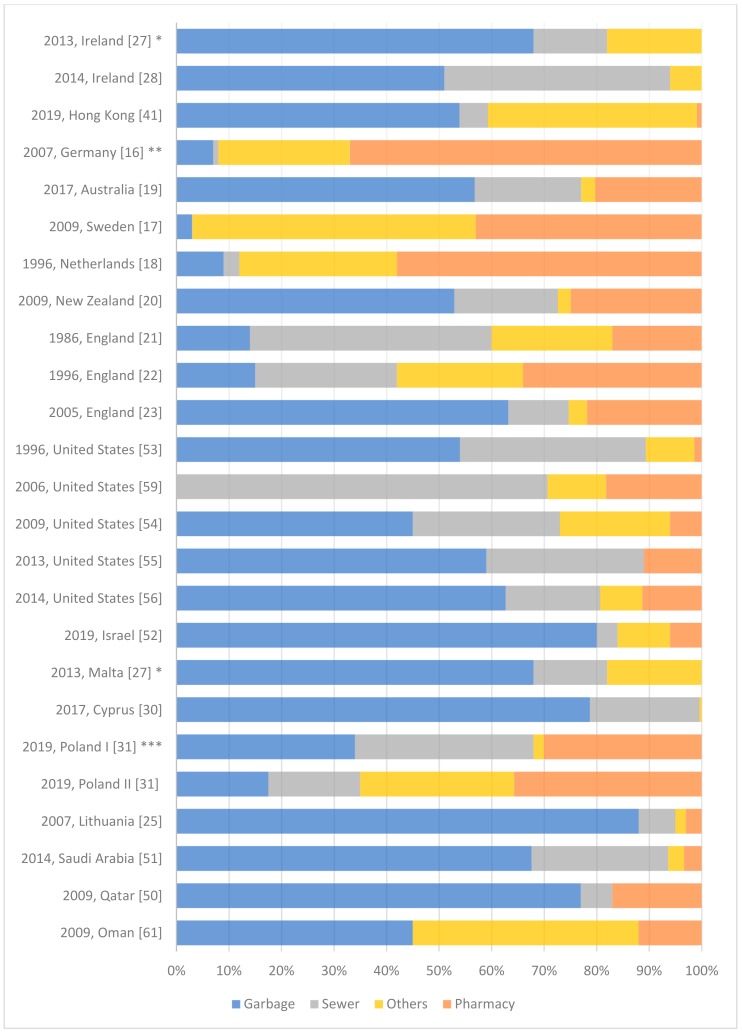

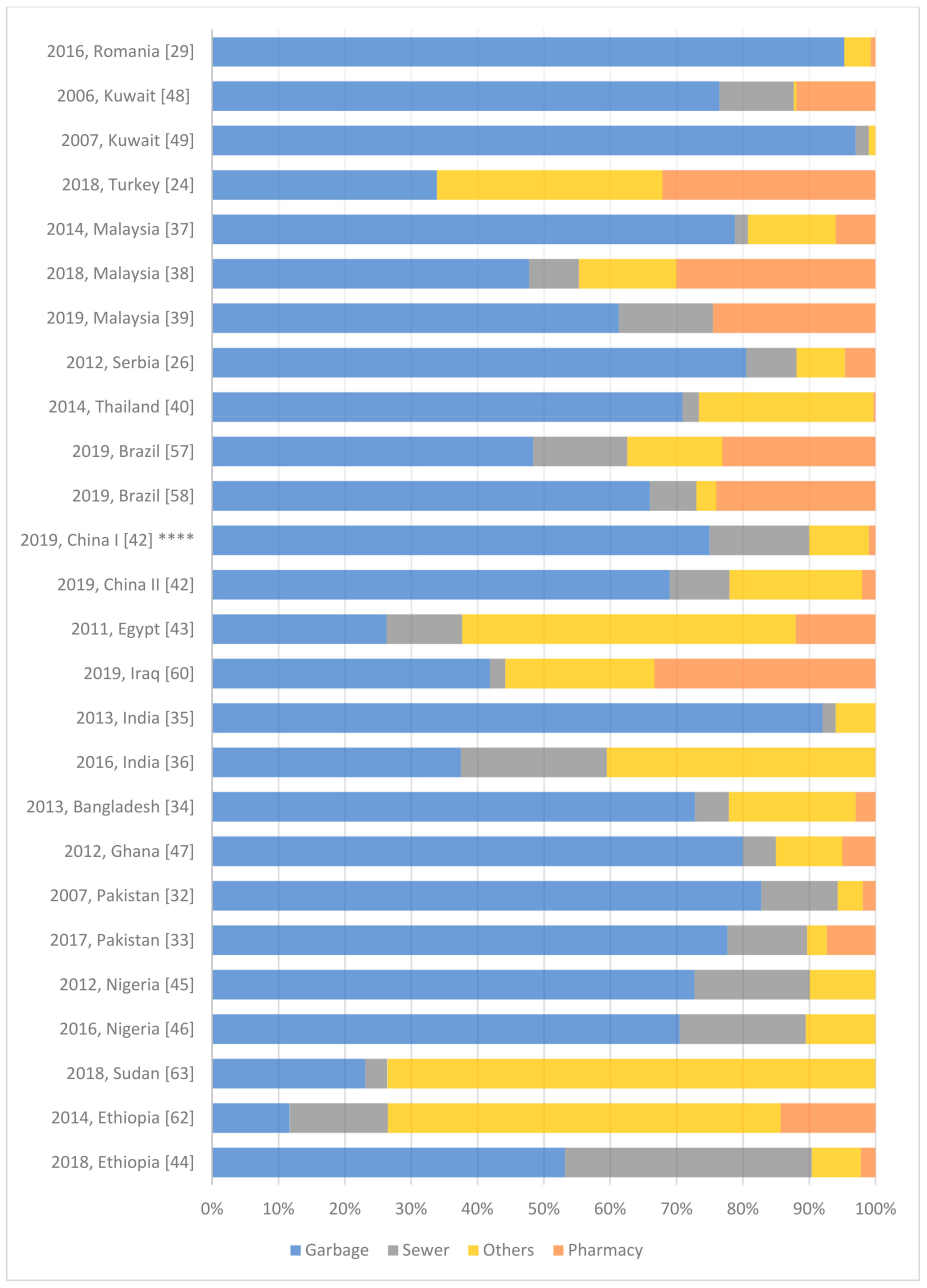
Disposal practice of unused medication among people as reported by peer-reviewed literature. * the survey was conducted in two countries (Ireland and Malta), and results were presented for both countries together. ^**^ many of the respondents (38.0%) dispose their medications in the recycling places and toxic waste bins, which considered as eco-friendly routes and combined with the pharmacy answers. *** two surveys were carried out: Survey I concerned households; Survey II focused on pharmacies. **** the study was conducted over two samples (Young-Adult I and Elderly II).

Figure 1 illustrates and summarizes the disposal practice of unused medication among people worldwide as reported by peer-reviewed literature (more details are available in Supplementary Materials S1). Countries were classified according to the Human Development Index (HDI), which is defined, by the United Nation, as “a summary measure of average achievement in key dimensions of human development: a long and healthy life, being knowledgeable and have a decent standard of living”. The HDI is the geometric mean of normalized indices for each of the three dimensions.

According to the results recorded in the surveys conducted in three Western and Northern European countries, participants return unused medicines to the pharmacy such as Germany (29%), Sweden (43%), and Netherland (58%) [16,17,18]. Additionally, the studies conducted in Australia (23%), New Zealand (24%), and England reported that about a quarter of the participants return their unused pharmaceuticals to the pharmacy [19,20,21,22,23].

Intriguingly, a private communication company in Turkey—Turkcell Global Bilgi (TGB)—provided a training activity (a symposium, a distant learning course, and informative materials) for its employees to raise awareness regarding the appropriate administration of medicine. A survey, then, was conducted to evaluate the approaches of the company employees regarding drug disposal. The internal campaign within the company seemed to present favorable changes in the behavior of its employees, since, most of the participants (66.1%) return unused medicines to the pharmacy or to the drug-box of the company [24]. 

Furthermore, disposing expired medications in the garbage is the most commonly reported practice in various studies conducted in England [23], Lithuania [25], Serbia [26], Malta [27], Ireland [27,28], Romania [29], Cyprus [30], Poland [31], Pakistan [32,33], Bangladesh [34], India [35,36], Malaysia [37,38,39], Thailand [40], Hong Kong [41], China [42], Egypt [43], Ethiopia [44], Nigeria [45,46], Ghana [47], Kuwait [48,49], Qatar [50], Saudi Arabia [51], Israel [52], United States [53,54,55,56], Brazil [57,58], New Zealand [20], and Australia [19].

Moreover, flushing unused medications down the toilet or sink, especially liquid dosage forms, has been reported in a lot of studies from a number of countries such as United States [53,54,55,59], England [21,22,23], New Zealand [20], Bangladesh [34], Iraq [60], and Saudi Arabia [51]. In contracts, the sewer method of disposal was less common in Sweden [17], Romania [29], or Oman [61].

In Ethiopia and Sudan, the surveys revealed that the most common disposal practice among people was to burn unused pharmaceuticals [62,63].

Intriguingly, if the disposal through garbage, sewer or burn is an eco-unfriendly route, the corresponding average of such hazardous practices would be around 72.7% ± 20.5% considering all reported studies. Therefore, it is relevant to analyze the habits of the population considering the HDI of the country, the Figure 1 shows a tendency of highly developed countries to recycle more unused medication rather than throwing or flushing them. Apart from this, some exceptions are notable such as Ireland, Iraq and Ethiopia where the percentage of eco-friendly disposal is not in agreement with their HDI. 

An analysis of disposal practices in terms of age and socioeconomic status could offer a deeper understanding of the problem and could help to plan action to encourage people for a proper medical disposal. Conducting such comparisons, however, needs a standardizing of survey form. In the survey that was conducted in Saudi Arabia, for instance, most of the respondents (93.2%; *n* = 1348) were males. There is, also, an absence of socioeconomic status in many studies. In addition, some of surveys were not conducted in public, but in specific places such as pharmacies (in Iraq) [60] or companies (in Turkey) [24].

### 3.2. Local Survey at the Saarland University

The participants were 64.7% female and 35.2% male. University students were 81.7% of the participants, while 18.3% were non-university students. Hence, most of the participants were of young age (52.2% were between 18 and 24 years old), and 32.9% of the participants were between 25 and 44 of age, and the remaining (14.7%) were over 45 years old. Only 17% of the respondents reported a daily use of prescription drugs, while the majority (77.2%) answered about their drugs consumption “occasionally when needed”. Most of the respondents (76.1%) kept a surplus of unexpired prescription drugs until expiration, and 11.3% donated them. More than half of the respondents (54.5%) seemed to be unfamiliar with the concept of drug-take-back systems, whilst few participants (6.8%) used this system.

**Figure 2:**
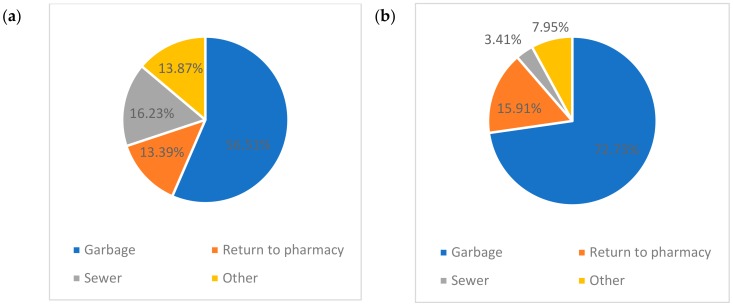
(**a**) Disposal methods according to our local survey. (**b**) The average of the disposal methods according to the results recorded in the 48 reviewed surveys.

Interestingly, as Figure 2a demonstrates, 72.7% of the participants disposed of expired prescription drugs in the garbage, while only 15.9% returned them to the pharmacy, and 3.4% disposed of the drugs down the toilet. Furthermore, 2.8% continued to use them after expiration.

A statistical test (Chi-squared test) was applied to assess if there is a difference in disposal practices between males and females, or between participants with different educational levels, results showed that there is no difference in disposal practices between these categories. It worthy to note, however, that our sample is small (*n* = 88) and not representative of the society (81.7% students) from a statistical point of view.

Assuming that the disposal route via garbage or sewer as eco-unfriendly route, the percentage of eco-unfriendly disposal practice is 76.1%. This percentage agrees with the average of eco-unfriendly disposal method (72.7%) calculated considering all reviewed studies (Figure 2b).

### 3.3. Regulations

Nowadays, in the era of growing pharmaceutical industries, putting forward and adopting legislation to monitor and control the disposal of expired medication is an urgent need. In effect, there is an absence of a global comprehensive and binding approach. There seem to be general recommendations grounded by global regulatory bodies, though still the handling and implementation differ on country bases. In this section, some of these essential recommendations and regulations is highlighted.

#### 3.3.1. Guidelines of World Health Organization (WHO)

Since World War II, the focus has shifted to the context of “environmental issues”. In 1972, 113 countries participated in the first United Nations (UN) conference held to address the aching international environmental issues. That year also witnessed the birth of the first environmental law, which aim was “to regulate human actions that may cause direct or indirect damage to the environment [64].” Later, in 1999, the WHO published guidelines regarding the safe disposal of unused pharmaceuticals [11]. These guidelines can be summarized as follows: (I) return to donor or manufacture; (II) high temperature incineration (greater than 1200 °C); (III) immobilization by waste encapsulation; (IV) chemical decomposition, if chemical expertise and materials are available.

#### 3.3.2. Guidelines of European Union (EU)

Nowadays, EU legislation obliges the member states to dispose of unused pharmaceuticals through proper methods. Still, a level of vagueness and ambiguity remains on how to enforce and conduct this task. In the case of Belgium, Italy, Greece, and Norway, for instance, returning unused medications to the pharmacy is mandatory and required by law. While in Austria, Croatia, Hungary, Ireland, Latvia, Luxembourg, France and Portugal, unused pharmaceuticals can be taken back to the pharmacy or recycling facility. 

On a more local level, many municipalities in different cities provide special collection sites, like “Gręboszyce” in Poland, “Mältan” in Sweden, and “Luopioinen” in Finland. In Germany, unused pharmaceuticals are classified as “municipal waste”, meaning that they are incinerated, biologically treated, and then landfilled. This process allows the “safe” disposal of pharmaceuticals among another regular household waste. Moreover, the private sector developed companies and associations responsible for the collection, management, and recycling of medications and pharmaceutical waste, such as “WasteServ Malta Ltd“ in Malta [65]. Another example of non-governmental associations for pharmaceutical waste management is running successfully in France, the process of collection and management of pharmaceutical waste has been proposed by several associations: LEEM, Adelphe and Cyclamed. In fact, these administrations designed new pictograms to explain the unused drug waste management to patients. They signed an agreement with the French Ministry of health that encourages industrials to feature these logos on the boxes or the instructions leaflets. This allows patients to understand how and where they can recycle their unused medications. They also started a commercial campaign throughout community pharmacies [66].

#### 3.3.3. Guidelines of U.S. Food and Drug Administration (FDA)

In 2017, the FDA published guidelines for safely disposing of medication in the home [67]. These guidelines can be briefly summarized as follows: (I) mix medications (do not crush tablets or capsules) with a bad-tasting substance; (II) place mixture in a container such as a sealed plastic bag or nondescript container; (III) throw the bag or container in the household trash; (IV) before disposing, scratch out personal information on medicine packaging; (V) If the above options are not viable, certain drugs can be safely flushed (ask your healthcare provider); (VI) check with your healthcare provider, pharmacist, or police department about public disposal locations or take-back events in your community.

#### 3.3.4. Guidelines of Australia

The Therapeutic Goods Administration (Department of Health, Australian Government) has published, in 2019, instructions about safe disposal of unwanted medicines as consumer information and education. It could be summed up as follows: (I) unsafe disposal of unwanted medicines can lead to environmental harm; (II) your local community pharmacy provides a free and convenient way to dispose of your unwanted medicines responsibly; (III) prescription medicines can be returned to your community pharmacy for free, safe disposal; (IV) most medicines can be placed directly in the disposal bin provided by the Return Unwanted Medicines (RUM) Project [68].

### 3.4. Local Waste Management Strategies

There are different strategies for managing solid waste around the world. Landfills, for instance, where the waste is buried, are the cheapest and the most common practice for solid waste management [69]. Open dumping is also a common practice, where solid waste is dumped on swamplands and low-lying areas [70]. In addition, burning and incineration methods are, globally, still common waste management practices, despite their destructive impact on the ecosystem dependent on several factors, especially the burning temperatures [71]. Solid waste recycling is an advanced technology which effectively turns waste into resources or provides an appropriate disposal approach [72]. Table 1 summarized the solid waste disposal methods per continent [73].

In the United States, municipal solid waste landfills are regulated by strict federal regulations. A study conducted in the U.S. reported that the disposal of unused medicines in landfills effectively eliminates the leakage of active pharmaceutically ingredient (APIs) to surface water. The landfills were shown to be able to retain more than 99.9% of APIs permanently [74].

The EU applies strict regulations to govern solid waste landfilling. The EU in their “Handbook on the Implementation of EC Environmental Legislation” addressed this issue as follows: “waste management strategies must aim primarily to prevent the generation of waste and to reduce its harmfulness. Where this is not possible, waste materials should be reused, recycled or recovered, or used as a source of energy. As a final resort, waste should be disposed of safely (e.g., by incineration or in landfill sites)” [75]. In effect, the negative impact on the environment is steadily controlled and reduced in some instances. 

In contrast, in many Asian, African, and South American countries, the landfills are poorly operated and regulated. These countries feature large populations; hence, the improper disposal of medications poses a serious global threat to the ecosystem. 

The worst effect of garbage disposal on the environment can be expected in countries where rubbish landfilling and open dumping are predominant and not properly regulated. In the case of uncontrolled landfilling and open solid dumping, the entry of the pharmaceuticals into the aqueous environment is simply postponed [20], as the contamination of neighboring surface or ground waters is inevitable. A study conducted by testing groundwater samples, from 23 points at nine distances from “Grindsted Landfill“ in Denmark, reported a presence and distribution of 13 pharmaceutical organic compounds through landfill leachates, such as sulfanilic acid and propyphenazone [76]. These solid waste landfills or dumps represent a substantial source of harmful pharmaceutical emissions into the ecosystem, especially with the absence of any control. India, for instance, with a population of 1.35 billion, was reviewed through a study revealing that, the predominant method for the disposal of unused pharmaceuticals is 92% via the garbage. Most of the solid waste generated remains uncollected, and in open lands [77].

The burning of waste materials which is adopted in many countries is not a completely environmentally friendly method. Most of pharmaceutical compounds are organic substances requiring sufficient oxygen, time, and temperature for ultimate incineration [71]. Thus, burning such pharmaceutical waste at low temperatures in open dumps is environmentally unfavorable and produces toxic air pollutants—e.g., aromatic compounds—especially since many pharmaceuticals contains halogens in their respective structure [78]. Dietary supplements, for example, also contain heavy metals such as iron, zinc, manganese, selenium, and molybdenum which do not incinerate easily. Ideally, the burning of pharmaceutical waste should be precisely controlled, otherwise the remains from the burning sites still have the tendency to be discharged into the ecosystem where they may cause significant damage [79].

## 4. Discussion

Nowadays, the world is facing massive pharmaceutical waste pollution, which represents a major ecological issue. Commercialized drugs can end up in the environment through diverse routes such as medication production and waste management, livestock, pet or human metabolization, the excretion of chemicals or even the inappropriate recycling of unused or expired medication [80]. A massive volume of pharmaceuticals has been found in the environment in soils and waters [81]. Even with those pieces of evidence, it is still difficult to assess the real impact or side effects those contaminants have on health. It is almost obvious, however, that releasing chemical substances in the ecosystem will have a dramatic effect in the future by enhancing pollution and global warming. In general, the ecological situation is getting worse and worse and precautionary behavior regarding drug disposal should be adopted for ethical purposes.

The present data collected from literature clearly illustrates that people around the world still lack awareness of appropriate disposal practices. These results shed a singular light on important facts regarding current and also popular methods of handling pharmaceutical waste. 

Whilst flushing unused pharmaceuticals down the sewer or throwing them in the garbage, for instance, are globally common disposal practices, they result in a risky and detrimental leakage of pharmaceuticals into the environment. The sewer route results in the presence of these active ingredients in the aqueous environment directly. Moreover, the detrimental effect of the garbage route on the environment varies, depending on the solid waste management strategy of each country, whether it will be burned, landfilled, or collected in open dumps. 

One eco-friendly approach to handle unused medications is called the drug take-back program (DTP). It is a program established in many developed countries by official agencies or local communities. This program aims to prevent pharmaceuticals from entering the environment improperly by providing a “safe” method of disposing of drugs. The current study, however, reveals an absence of a systematic application of the Drug Take Back Program in several countries.

Above all, these findings obtained from reviewed literature are corroborated by our local survey. Therefore, there exists an obvious urgency for increasing public awareness on the issue of medication disposal, the impact on the environment and health, and exploring means to lessen the consequences of the issue. Awareness-raising sessions that aim to improve proper medical disposal should be planned, with a special focus on the senior population as a target group. The complexity of medications should be considered in the elderly, where deprescribing or stopping medication is frequent, leading to the accumulation of unused drugs. It was reported that patients aged 65 years and older in the USA use 40% of all prescription drugs [82]. Surveys about disposal medication should be encouraged to gather more information that allow to plan actions for increasing public awareness. The form of our local survey (Supplementary Materials S2) could be a recommended form for conducting more surveys in many other countries.

Besides, with regard to some reported global waste management strategies, undoubtedly, it is clear that the current global handling of pharmaceutical wastes could cause enormous environmental damage via undesirable leakage of pharmaceuticals into the ecosystem, necessitating the urgent need to adopt particular legislation to control the disposal of expired medications.

In the light of the above, expired medications consist a large amount of pharmaceuticals waste and it seems like the current understanding of their concept, it is counterproductive to the purpose of wellbeing. 

The World Health Organization (WHO) defines the expiry date as follows: "the date given on the individual container of a pharmaceutical product and including the date on which the product is expected to remain within the specific action, if stored correctly. It is established for each batch by adding the shelf-life to the date of manufacture.” [83]. The United States Food and Drug Administration’s (USFDA) definition reads: “the date placed on the container/labels of an API designating the time during which the API is expected to remain within established shelf-life specifications if stored under defined conditions and after which it should not be used." [84]. 

The administration of a medication which passed its expiration date, however, does not necessarily indicate inevitable inefficacy or even toxicity. Apart from some cases, many drugs generally remain effective and safe even beyond their expiration dates, which indicates that expiry dates deserve a closer inspection [85]. 

The expiration date is usually designated by the manufacturer. In fact, it is the final day on which the manufacturer of the medication guarantees fully its quality and safety, and this date is determined by stability testing. Hence, the expiration of this guarantee does not necessarily imply that the drug will be somewhat inefficient or even toxic after this specific date. Rather, the chemical composition has taken a step into the unknown beyond the date established by the stability test. The issue with the management of expired medications stems from the lack or even absence of knowledge regarding the chemical composition, hence quality and safety after this date.

After passing the expiration date, a drug’s chemical composition is left to chances related to its stability, which is tied to handling and storage conditions. The drug enters a hibernation state (purple) in terms of its chemical epistemic capacities. Is the drug still valid (blue)? Has the drug decayed and decomposed beyond retreat (red)? An action is required to investigate the epistemic state of purple medications.

In an attempt to appraise the underlying epistemic capacities of the chemical composition of medications; specific colors will be assigned. The term “Blue Medication” would refer to the drug which is within the period of validity before the expiration date. “Red Medication” would refer to the drug which passes its expiration date and undergoes degradation. Between blue and red areas there is the medication which passes its expiration date and its stability is unknown. It has two possibilities at the same time before being tested, which are degradation or validation. It is more logical to define it as “Purple Medication” instead of “expired”. Reassessing the chemical composition by conducting stability tests on purple medications exemplifies an appropriate tipping point to distinguish true “waste” from remaining “value”.

Bayer® Aspirin, for instance, typically has an expiration date up to three years after manufacture. A report has shown, however, that Bayer® Aspirin remains valid up to five years [85]. Empirically, this specific medication proved to be blue (valid) for two extra years; after passing the expiration date, it becomes purple (unknown), yet, theoretically, it was assigned a reduced and lessened utility period, and labeled red (expired) after just three years. Furthermore, a study conducted on eight long-expired medications (28 to 40) years containing 14 APIs, demonstrated that 12 compounds out of the 14 were at least 90% still intact [85].

A waste of pharmaceuticals is, to some extent, a waste of ethics. According to a report by the UN, more than 1.5 million people died in Africa in 2015 due to preventable or treatable diseases with affordable, yet locally unavailable medicines [86]. In contrast, tons of unused medicines are thrown or burnt every year in developed countries. Since these medicines offer hope to the sick and dying. There have been attempts to render the waste of one party into the means of survival of others. 

Several organizations published guidelines for drug donation in the 1980s. In 1990, for instance, an emergency shipment of medical assistance was sent to South Sudan. All the drugs were labeled in French, which is not commonly spoken there. In addition, most of the medications did not meet the recipients’ needs in a country devastated by war. The shipment included expired antibiotics, hypercholesterolemia drugs, X-ray solutions, and contact lens solutions [87]. Moreover, in 1991, Pharmacists Without Borders collected four million kilograms of unused medications from 4000 pharmacies in France for international aid programs. They found only 20% of the collected drugs could be used, and 80% had to be burned. It is believed, according to a report by WHO, that the reason behind such high percentage of deterioration is due to poor handling and storage conditions [87].

In 1986, the Shelf Life Extension Program (SLEP) was established by both the U.S. Department of Defense (DoD) and the USFDA. It was a federal program aiming to save government resources by extending the shelf-life of medications in military stockpiles. More than 3000 batches of medications (122 different drug products) were tested by the FDA, and approximately 90% were valid for use after the expiration date. Consequently, the average extension period was 5.5 years, and some of the batches were extended by more than 20 years. The expiration date of Naproxen tablets, for instance, was extended for an average of 4.5 years. The expiration date of calcium chloride Injection-solution also was extended up to about 7 years [88].

The Shelf Life Extension Program represents a successful strategy for managing the American Army medical resources. Still, such programs cannot tackle the global problems related to pharmaceutical waste because this program is private to special government agencies and not available to the wider public. One reason is that all medications in military stockpiles are preserved in ideal storage conditions, while these conditions are not available in the case of randomly collected drugs for the public.

By and large, the drug take-back program aims to reduce environmental contamination by providing a relatively safe disposal of expired medications upon collecting from the public. Yet, the critical fault of such programs is throwing the collected medications in the incinerator, which enables pharmaceuticals to enter the environment. Thus, reducing or postponing their destructive entrance to the environment and preventing twofold economic loss is an urgent priority [89]. The lack of innovation to enhance proper medical disposal methods is a major challenge. One answer could be the use of mathematical modeling to stimulate the improvement of recycling logistics networks at better costs [90].

Medication recycling programs, as an alternative to incineration, could bear a high economic effectiveness. This is of particular importance in the case of a medication which has an expensive API. One tablet of ZOFRAN (Ondansetron), for instance, costs around USD 24 [91]. Theoretically, the API can be extracted and reemployed in a new formulation to render it of “value” again. Such an approach could be a new revolution in the field of pharmacy, by creating an opportunity for new recycling companies which are different from synthetic companies. Their main duties would revolve around extracting, purifying, and repacking. Although such programs could be losing out in economic terms, it would be an environmentally beneficial strategy.

Finally, a limitation of this study was the limited number of countries covered in reviewed surveys. In addition, it should be pointed out that results recorded in the reviewed surveys might vary depending on different factors, such as the year of the study, demographical characteristics of participants, and their social environment. There is a need for conducting more surveys in many other countries. Besides, these studies need to be updated continuously to evaluate changes in public awareness. A standardization of the questionnaire is also essential to facilitate the comparison of practices by the public around the world. This paper, however, points out the need for more research regarding medical waste management.

## 5. Conclusions

The excessive production, prescriptions, and consumption of pharmaceuticals has led to a vast volume of expired medications which have multifaceted harmful impacts, especially to the environment. Noteworthily, the issue of expired medications is an interrelated problem being contributed by all parties involved in the production and consumption process of pharmaceuticals. On this basis, there is an urgent need to adopt a collaborative effort to address this issue by each of legislation authorities, pharmaceutical companies, and patients.

The issue of expired medications stems from the absence of particular legislation to control the disposal of expired medication. Currently, in the epoch of growing pharmaceutical industries, putting forward and adopting particular legislation to control the disposal of expired medication is an urgent must.

Pharmaceutical companies, for their part, need to take greater responsibility of the life cycle of their products. They should, first, consider the balance between production and consumption. In addition, an extension of the expiration date of a drug, utilizing innovative stability testes, would help in reducing the annual volume of expired medications. Furthermore, most of them ought to mention the instructions of proper disposal practices on the package, for instance “please contact your local drug take-back program for the disposal of this product.” This simple mention would be of great contribution to raising public awareness regarding the appropriate disposal practices.

Finally, the present data collected from the literature illustrate, undoubtedly, that participants indicating a significant lack of public awareness regarding the appropriate disposal practices, it demonstrates also an absence of the Drug Take Back Program in several countries. Therefore, there is a need to make Drug Take Back Program more popular and effective. Public awareness campaigns would also be an action towards the raising wider public awareness of this “side effects” of pharmacy and pharmaceuticals on the wider human society and the environment. 

## Figures and Tables

**Table 1:** Solid waste disposal methods per continent. According to the Global Development Research Center [73].

Continent	Landfill	Dump	Recycle	Burn	Other
Asia	30.9%	50.9%	8.5%	6.4%	4.5%
Africa	29.3%	47.0%	3.9%	10.6%	8.4%
Europe	27.6%	33.0%	10.7%	25.6%	4.4%
South America	60.5%	34.0%	3.2%	7.5%	2%
North America	91.1%	0%	8.1%	0%	0%

## Supplementary Materials

The following are available online at https://www.mdpi.com/1660-4601/17/3/787/s1, Supplementary Material S1: Disposal practice of unused medication among people as reported by peer-reviewed literature, Supplementary Material S2: Questionnaire about medication disposal practices.
