# Supplementary material for: Expired Medication: Societal, Regulatory and Ethical Aspects of a Wasted Opportunity
Source: Int J Environ Res Public Health. 2020 Jan 27;17(3):787. doi: 10.3390/ijerph17030787 (PMC7037917; doi:10.3390/ijerph17030787)
Supplement: Supplementary file 1 [file ijerph-17-00787-s001.pdf]

Supplementary Material I. Disposal practice of unused medication among people as reported by peer-reviewed literature ([A] respondents could select more than one answer).

Author (s)	Year	Locality	Sample (n)	Method of Disposal				Environmental
				Garbage	Toilet or sink	Return to pharmacy	Other (stated)	Eco-friendly
Ridout et al. [21]	1986	England	443	14.0%	46.0%	17.0%	15.0% (kept medicines)<br>7.0% (give to others)	39.0%
Blom et al. [18]	1996	Netherlands	2154	9.0%	3.0%	58.0%	16% (chemical waste)<br>6% (kept medicines)	80.0%
Kuspis and Krenzelok [53]	1996	United States	500	54.0%	35.4%	1.4%	7.2% (kept medicines)<br>2% (finished medications)	10.6%
Sullivan and George [22]	1996	England	400	15.0%	27.0%	34.0%	15% (kept medicines)	49.0%
Bound and Voulvoulis [23]	2005	England	392	63.2%	11.5%	21.8%	3.5% (waste sites or another disposal facility)	25.3%
Abahussain et al. [48]	2006	Kuwait	300 [A]	76.5%	11.2%	11.9%	8.5% (give to friend)	20.4%
Seehusen and Edwards [59]	2006	United States	301 [A]	-	53.80% (toilet)<br>35.20% (sink)	22.9%	14% (returned to healthcare provider)	36.9%

**Author (s)**	**Year**	**Locality**	**Sample Size (n)**	**Method of Disposal**				**Environmental**
				**Garbage**	**Toilet or sink**	**Return to pharmacy**	**Other (stated)**	**Eco-friendly**
Abahussain et al. [49]	2007	Kuwait	200	97.0%	2.00%	-	0.5% (give to friend)	<1.0%
Gotz and Keil [16]	2007	Germany (solid medication)	1306	7% always 9% usually 14% sometimes 13% rarely	1% always 2% usually 7% sometimes 6% rarely	29% always 11% usually 15% sometimes 11% rarely	23% recycled alongside cardboard and plastics 15% toxic waste bins	67.0%
		Germany (liquid medication)			10% always 8% usually 13% sometimes 12% rarely			-
A. Ahmed et al. [32]	2007	Pakistan	1022	82.8%	11.5%	1.9%	3.8% (do not know)	1.9%

Author (s)	Year	Locality	Sample Size (n)	Method of Disposal				Environmental
				Garbage	Toilet or sink	Return to pharmacy	Other (stated)	Eco-friendly
Krupiene and Dvarioniene [25]	2007	Lithuania (towns)	200	88.0%	7.0%	3.0%	2% (burning)	3.0%
		Lithuania (suburbs)		87.0%	6.0%	-	12.5% (burning)	0.0%
		Lithuania (countryside)		50.0%	-	-	50% (burning)	0.0%
Braund et al. [20]	2009	New Zealand (liquid)	452	24.0%	55.0%	17.0%	0.7% (give away or burn)	17.0%
		New Zealand (tablets and capsules)		51.0%	19.0%	24.0%	2.4% (give away or burn)	24.0%
		New Zealand (ointments and creams)		80.0%	<1%	13.0%	2.4% (give away or burn)	13.0%

Author (s)	Year	Locality	Sample Size (n)	Method of Disposal				Environmental
				Garbage	Toilet or sink	Return to pharmacy	Other (stated)	Eco-friendly
Kotchen et al. [54]	2009	United States	1005	45.0%	28.0%	6.0%	5% (hazardous waste center)<br>12% (store at home)<br>4% (unspecified method)	23.0%
Persson et al. [17]	2009	Sweden	1000	3.0%	-	43.0%	55% (kept medicines)	97.0%
Abdo-Rabbo et al. [61]	2009	Oman	6675	45.0%	-	12.0%	41% (keep for future use)	53.0%
Kheir et al. [50]	2011	Qatar	49	77.0%	6.0%	-	16% (kept medicines)	16.0%
M. El-hamamsy et al. [43]	2011	Egypt	316	26.3%	11.4%	12.0%	15.5% (Use them before expiration)<br>11.4% (Don't dispose)	39.5%
Auta et al. [45]	2012	Nigeria	240	72.7%	12.9% (toilet)<br>4.5% (sink)	-	9.9% (burn)	0.0%

**Author (s)**	**Year**	**Locality**	**Sample Size (n)**	**Method of Disposal**				**Environmental**
				**Garbage**	**Toilet or sink**	**Return to pharmacy**	**Other (stated)**	**Eco-friendly**
Sasu et al. [47]	2012	Ghana	83	>80%	<5%	<5%	>10% (burn)	<5.0%
M. Kusturica et al. [26]	2012	Serbia (Urban)	208	85.6%	8.7%	4.8%	1% (burn or give to a friend)	4.8%
		Serbia (Rural)		74.5%	6.4%	4.3%	14.9% (burn or give to a friend)	4.3%
Iabu et al. [34]	2013	Bangladesh (liquid)	290	17.2%	58.2%	1.0%	22.7% (give away or no answer)	24.6%
		Bangladesh (solid)		72.8%	5.1%	3.0%	17.2% (give away or no answer)	22.1%
		Bangladesh (semisolid)		33.1%	16.2%	11.0%	37.9% (give away or no answer)	50.7%

Author (s)	Year	Locality	Sample Size (n)	Method of Disposal				Environmental
				Garbage	Toilet or sink	Return to pharmacy	Other (stated)	Eco-friendly
C. Fenech et al. [27]	2013	Malta and Ireland (liquid)	1130	57.0%	28.0%	<10%	-	<10%
		Malta and Ireland (pills)		68.0%	14.0%	-	-	0.0%
S. Aditya and H. Singh [35]	2013	India (solid)	244	92.0%	2.0%	-	-	0.0%
		India (semisolid)		94.0%	-	-	-	0.0%
		India (liquid)		74.0%	21.0%	-	-	0.0%
S. Wieczorkiewicz et al. [55]	2013	United States	445	59.0%	30.0%	11.0%	-	11.0%
O. Fatokun [37]	2014	Malaysia	250	78.8%	2.0%	6.4%	12.8% (fridge, give to a friend, leftover)	19.2%

Author (s)	Year	Locality	Sample Size (n)	Method of Disposal				Environmental
				Garbage	Toilet or sink	Return to pharmacy	Other (stated)	Eco-friendly
T. Atinafu *et al* [62]	2014	Ethiopia	384	11.7%	14.8%	14.3%	25.3% (burn) 11.2% (bury) 2.1% (donate) 20.6% (others)	16.4%
A. Vellinga *et al*. [28]	2014	Ireland	398	51.0%	43.0%	-	6.0%	0.0%
Q. Abdallah *et al*. [51]	2014	Saudi Arabia (liquid)	1386	71.7%	20.0%	5.0%	2.8%	5.0%
		Saudi Arabia (solid)		63.4%	31.9%	1.7%	1.9%	1.7%
Arkaravichien *et al*. [40]	2014	Thailand (liquid)	311	64.6%	7.4%	0.0%	22.0% (never discarded)	22.0%
		Thailand (solid)		81.4%	0.0%	1.0%	1.6% (land filled) 32.8% (never discarded)	33.8%
		Thailand (semisolid)		66.6%	0.0%	0.0%	0.6% (land filled)	0%

**Author (s)**	**Year**	**Locality**	**Sample Size (n)**	**Method of Disposal**				**Environmental**
				**Garbage**	**Toilet or sink**	**Return to pharmac**	**Other (stated)**	**Eco-friendly**
A. Law et al. [56]	2014	United States (web)	238 [A]	62.7%	18.0%	11.3%	17.4% (store)	28.7%
		United States (paper)	68	-	4.3%	1.8%	8% (unspecified)	1.8%
S. Banwat et al. [46]	2016	Nigeria	105	70.5%	19.0%	-	10.5% (burn)	0.0%
D. Tit et al. [29]	2016	Romania	771	95.3%	-	0.7%	4% (other places)	0.7%
M. Kozak et al. [36]	2016	India	200	37.5%	22.0%	-	40.5% (store home)	40.5%
M. Bashaar et al. [33]	2017	Pakistan	301	77.7%	12.0%	7.3%	1.3% (donate)	8.4%
E. Bettington [19]	2017	Australia	2521 [A]	64.4%	23.0%	23.0%	3% (burn)	23.0%
A. Zorpas et al. [30]	2017	Cyprus	184 [A]	92.4%	24.5%	-	0.5% (burn)	0.0%
K. Addin et al. [63]	2018	Sudan	320	23.1%	3.3%	-	48.9% (Burn)	0.0%

Author (s)	Year	Locality	Sample Size (n)	Method of Disposal				Environmental
				Garbage	Toilet or sink	Return to pharmacy	Other (stated)	Eco-friendly
A. Yang et al. [38]	2018	Malaysia	148	47.8%	7.5%	30.0%	5.1% (keep) 4.7% (donate) 1.6% (burn) 3.2% (others)	39.8%
A. Akici et al. [24]	2018	Turkey	1121	33.9%		34.0%	32.1% (bring to drug-box of the company)	66.1%
Y. Ayele et al. [44]	2018	Ethiopia	694	53.2%	37.2%	2.2%	0.4% (donate) 7.0% (others)	2.6%
Barnett et al. [52]	2019	Israel	602	80.0%	4.0%	6.0%	-	6.0%
G. Viana et al. [57]	2019	Brazil	182	48.4%	14.2%	23.1%	8.2% (keep) 2.2% (landfill) 3.8% (others)	31.3%
S. Chung et al. [41]	2019	Hong Kong, China	1865	53.9%	5.4%	0.9%	0.5% (burn) 0.5% (donate) 0.6% (sell) 35.3% (use)	37.3%

**Author (s)**	**Year**	**Locality**	**Sample Size (n)**	**Method of Disposal**				**Environmental**
				**Garbage**	**Toilet or sink**	**Return to pharmacy**	**Other (stated)**	**Eco-friendly**
G. Quadra et al. [58]	2019	Brazil	540	66.0%	7.0%	24.0%	2.0% (share)<br>1.0% (burn)	26.0%
K. Albaroodi [60]	2019	Iraq (semisolid)	129	41.9%	2.3%	33.3%	21.7% (burn)<br>0.8% (other)	33.3%
		Iraq (liquid)		16.3%	60.5%	19.4%	2.3% (burn)<br>1.6% (other)	19.4%
J. Rogowska [31]	2019	Poland (survey I)	450	68.0%		30.0%	-	30.0%
		Poland (survey II)	635	35.0%		35.7%	29.3% (other)	35.7%
Ariffin et al. [39]	2019	Malaysia	103[A]	63.1%	14.6%	25.2%	-	25.2%
X. Yu et al. [42]	2019	China (Young-adult)	365	75.0%	15.0%	1.0%	9.0% (burn)	1.0%
		China (elderly)	206	69.0%	9.0%	2.0%	20.0% (burn)	2.0%

Supplementary Material II

Questionnaire

Dear participant,

Thank you for your participation. This questionnaire is designed to gather information for a research project about common practices related to the disposal of expired prescription drugs. Please answer the following questions freely and to the best of your knowledge. The questionnaire is anonymous and individual data will be kept strictly confidential. Further information: s8alfaez@stud.uni-saarland.de

1. Age
◎ 18-24 ◎ 25-34 ◎ 35-44 ◎ 45-55 ◎ Over 55

---

2. Gender
◎ Male ◎ Female ◎ Other / Unspecified

---

3. Level of education
◎ High school ◎ College (Abitur) ◎ University degree ◎ Unspecified

---

4. How often do you use prescription drugs?
◎ Every day ◎ A few times a week ◎ Occasionally when needed ◎ Never

---

5. What do you do with surplus *unexpired* prescription drugs?
◎ Dispose them ◎ Donate /share them ◎ Keep them until they expire ◎ Other

---

6. Please estimate which percentage of your own prescription medicines *expire* before you use them.
◎ None ◎ less than 25% ◎ less than 50% ◎ more than 50% ◎ more than 75%

---

7. How do you dispose expired prescription drugs?
◎ Down the toilet ◎ Down the sink ◎ Garbage ◎ Compost ◎ Burn
◎ In public (river, bin, forest) ◎ Return to pharmacy ◎ Use a drug-take-back system
◎ Continue to use medication even if it is expired ◎ No answer ◎ Other ________

---

8. Are you familiar with the concept of drug-take-back systems?
◎ No ◎ Heard of it ◎ Use it myself ◎ Would use it if available

---

9. In your personal opinion, how should *expired prescription drugs* be handled? (You can add any relevant comments here, cont. on the back)
____
____
